# Metformin and Weight Changes in Non-diabetic Older People With HIV (METFORAGING): A Double-Blind, Randomized, Placebo-Controlled, Pilot Trial

**DOI:** 10.1093/ofid/ofag489

**Published:** 2026-08-06

**Authors:** Cristina Marcelo-Calvo, Andrés Esteban-Cantos, Francisco Jurado, Rocío Montejano, Lucía Gutiérrez-García, Alejandro de Gea-Grela, Patricia Martínez-Martín, Alejandro Díez-Vidal, Rosa de Miguel Buckley, Carlos M Oñoro-López, Juan C González, Luz Martín-Carbonero, José I Bernardino, Rocío Menéndez Colino, Noemí González Pérez de Villar, Berta Rodés, José R Arribas

**Affiliations:** Department of Internal Medicine, HIV Unit, La Paz University Hospital, Madrid, Spain; Hospital La Paz Institute for Health Research (IdiPAZ), Madrid, Spain; CIBER of Infectious Diseases (CIBERINFEC), Madrid, Spain; Hospital La Paz Institute for Health Research (IdiPAZ), Madrid, Spain; CIBER of Infectious Diseases (CIBERINFEC), Madrid, Spain; Genetic and Molecular Epidemiology Group, Spanish National Cancer Research Centre (CNIO), Madrid, Spain; Department of Internal Medicine, HIV Unit, La Paz University Hospital, Madrid, Spain; Hospital La Paz Institute for Health Research (IdiPAZ), Madrid, Spain; CIBER of Infectious Diseases (CIBERINFEC), Madrid, Spain; Hospital La Paz Institute for Health Research (IdiPAZ), Madrid, Spain; Department of Internal Medicine, HIV Unit, La Paz University Hospital, Madrid, Spain; Hospital La Paz Institute for Health Research (IdiPAZ), Madrid, Spain; Department of Internal Medicine, HIV Unit, La Paz University Hospital, Madrid, Spain; Hospital La Paz Institute for Health Research (IdiPAZ), Madrid, Spain; CIBER of Infectious Diseases (CIBERINFEC), Madrid, Spain; Department of Internal Medicine, HIV Unit, La Paz University Hospital, Madrid, Spain; Hospital La Paz Institute for Health Research (IdiPAZ), Madrid, Spain; Department of Internal Medicine, HIV Unit, La Paz University Hospital, Madrid, Spain; Hospital La Paz Institute for Health Research (IdiPAZ), Madrid, Spain; CIBER of Infectious Diseases (CIBERINFEC), Madrid, Spain; Department of Internal Medicine, HIV Unit, La Paz University Hospital, Madrid, Spain; Hospital La Paz Institute for Health Research (IdiPAZ), Madrid, Spain; Hospital La Paz Institute for Health Research (IdiPAZ), Madrid, Spain; Department of Internal Medicine, HIV Unit, La Paz University Hospital, Madrid, Spain; Hospital La Paz Institute for Health Research (IdiPAZ), Madrid, Spain; CIBER of Infectious Diseases (CIBERINFEC), Madrid, Spain; Department of Internal Medicine, HIV Unit, La Paz University Hospital, Madrid, Spain; Hospital La Paz Institute for Health Research (IdiPAZ), Madrid, Spain; CIBER of Infectious Diseases (CIBERINFEC), Madrid, Spain; Hospital La Paz Institute for Health Research (IdiPAZ), Madrid, Spain; Department of Geriatrics, La Paz University Hospital, Madrid, Spain; Department of Endocrinology and Nutrition, La Paz University Hospital, Madrid, Spain; Hospital La Paz Institute for Health Research (IdiPAZ), Madrid, Spain; CIBER of Infectious Diseases (CIBERINFEC), Madrid, Spain; Department of Internal Medicine, HIV Unit, La Paz University Hospital, Madrid, Spain; Hospital La Paz Institute for Health Research (IdiPAZ), Madrid, Spain; CIBER of Infectious Diseases (CIBERINFEC), Madrid, Spain

**Keywords:** GDF-15, HIV, metformin, weight

## Abstract

**Background:**

Weight gain is a major complication of contemporary antiretroviral treatment (ART). We evaluated the effects of metformin on weight trajectories and metabolic outcomes in non-diabetic, non-obese, older persons with HIV-1 (PWH).

**Methods:**

In this double-blind, randomized, placebo-controlled pilot trial (METFORAGING), PWH 50 years or older, virologically suppressed, without diabetes or insulin resistance, were randomly assigned (1:1) to metformin 850 mg or placebo twice daily for 96 weeks at La Paz University Hospital (Madrid, Spain). This prespecified secondary analysis evaluated changes in body weight, body mass index (BMI), glycemic indices, lipid parameters, and serum growth differentiation factor-15 (GDF-15) in the per-protocol population.

**Results:**

Forty participants were randomly assigned (metformin n = 19; placebo n = 21); 35 completed treatment through week 96 (per-protocol population). At week 96, mean weight change was −1.5 kg (95% CI −2.9 to −0.03) with metformin and +1.3 kg (−0.7 to 3.3) with placebo (between-group difference −2.8 kg [95% CI −5.2 to −0.4]; *P* = .025). After discontinuation, weight in the metformin group returned toward baseline (between-group difference from week 96 to 144: 2.2 kg [95% CI 0.05 to 4.5]; *P* = .045). At week 96, changes in body weight correlated inversely with changes in serum GDF-15 in the metformin group (*r* = −0.67; *P* = .003). No serious adverse events were attributed to metformin.

**Conclusions:**

Metformin attenuated weight gain in nondiabetic, nonobese PWH receiving contemporary ART, but the effect was not sustained after discontinuation. These findings support larger trials to confirm efficacy and define optimal treatment duration.

**Trial Registration:**

EudraCT 2021-003299-15 (https://www.clinicaltrialsregister.eu).

## Teaser

Metformin attenuated progressive weight gain in nondiabetic, nonobese older persons with HIV over 96 weeks compared with placebo. The effect reversed after discontinuation and correlated inversely with serum GDF-15 changes, suggesting an exposure-dependent, GDF-15-mediated mechanism.

Contemporary antiretroviral regimens, particularly those including tenofovir alafenamide (TAF) and integrase strand transfer inhibitors (INSTIs) such as dolutegravir and bictegravir, have been associated with greater weight gain compared with regimens containing tenofovir disoproxil fumarate or nonnucleoside reverse transcriptase inhibitors, which have been linked to relative weight suppression or weight loss [1].

Excessive weight gain in PWH is associated with increased risk of type 2 diabetes mellitus, which in turn is linked to a higher risk of myocardial infarction, cardiovascular disease, and chronic kidney disease, with potential long-term effects on mortality [2]. Large cohort and longitudinal studies have consistently reported that weight gain is a key driver of metabolic syndrome, diabetes, and cardiovascular risk in PWH [3].

Management strategies to address ART-associated weight gain remain limited, and available evidence suggests that antiretroviral switching alone is unlikely to produce clinically meaningful weight reduction in most patients [4, 5]. Pharmacologic interventions such as GLP-1 receptor agonists can induce substantial weight loss in PWH [6], but benefits may not be sustained after discontinuation [7]. In addition, the high cost of GLP-1–based therapies and the need for long-term or indefinite treatment may limit their scalability and real-world implementation, particularly in resource-limited settings [8].

Metformin is a safe, widely available, and cost-effective agent traditionally used for type 2 diabetes mellitus, with a well-established long-term safety profile based on more than 6 decades of widespread clinical use. Beyond improving insulin sensitivity, metformin can reduce energy intake, and promote modest weight loss through complementary mechanisms, including suppression of hepatic gluconeogenesis, reduction of intestinal glucose absorption, and enhancement of satiety mediated in part by GLP-1 signaling and the GDF-15 axis [9, 10].

In non-HIV populations, a meta-analysis including both individuals with and without type 2 diabetes, metformin showed modest but consistent effects on body weight (mean BMI reduction ∼0.56 kg/m^2^)[11], with clinically meaningful weight loss (≥5%) achieved in approximately 25%–30% of treated individuals, a threshold considered relevant in obesity management guidelines [12]. In PWH, metformin was initially evaluated for lipodystrophy and metabolic complications, with early studies suggesting reductions in visceral adiposity, and insulin resistance [13]. Recent meta-analyses indicated consistent improvements in cardiometabolic parameters of nondiabetic PWH, without significant change in BMI [14]. A phase II randomized, placebo-controlled trial in Tanzania also reported significant weight reduction with metformin in PWH with prediabetes [15]. However, metformin has not been specifically evaluated as a strategy to prevent weight gain in nondiabetic, nonobese PWH receiving contemporary ART regimens that include INSTIs and TAF.

The METFORAGING trial was primarily designed to evaluate the effects of metformin on biological aging in PWH using DNA methylation-based epigenetic biomarkers as the primary endpoint [16]. In addition to these prespecified primary analyses, the trial also collected longitudinal metabolic data. Given the increasing clinical importance of weight gain and cardiometabolic complications in PWH receiving contemporary ART, we report here secondary analyses evaluating the effects of metformin on metabolic outcomes, with a particular focus on weight trajectories during metformin treatment, and after treatment discontinuation.

## METHODS

### Study Design

METFORAGING was a double-blind, randomized, parallel-group, placebo-controlled, pilot trial conducted at the HIV clinic of La Paz University Hospital (Madrid, Spain). Full details of the trial design, randomization procedures, and masking have been described in detail previously [16]. The primary endpoint was change in DNA methylation-based epigenetic biomarkers of biological aging, reported separately [16]. The present analysis reports prespecified secondary and exploratory metabolic outcomes.

Enrollment began on 2 March 2022. Participants were followed through week 144, including a 96-week intervention period followed by post-treatment observational follow-up without study medication. The protocol is available in the EudraCT registry (2021-003299-15).

### Participants

As previously reported [16], eligible participants were PWH aged 50 years or older, receiving stable ART for at least 3 months before screening, with sustained virological suppression (HIV-RNA <50 copies/mL) for at least 1 year before enrollment and a CD4+ T-cell count >500 cells/μL. Participants had no obesity (BMI <30 kg/m^2^), no diabetes or prediabetes (defined as fasting glucose 100–125 mg/dL, 2-hours glucose after a 75 g oral glucose tolerance test 140–199 mg/dL, or glycated hemoglobin (HbA1c) 5.7%–6.4%), and no insulin resistance (Homeostatic Model Assessment for Insulin Resistance (HOMA-IR) ≤ 2.6).

All participants provided written informed consent. The trial was approved by the Ethics Committee of La Paz University Hospital (reference number: HULP-5971), and sponsored by the La Paz University Hospital Research Foundation. The study was conducted in accordance with the Declaration of Helsinki and the International Council for Harmonisation Good Clinical Practice.

### Randomization and Masking

Participants were randomly assigned (1:1) to receive metformin or matched placebo using central computer-allocation, as previously described [16]. Participants, investigators, and outcome assessors were masked to treatment assignment.

### Interventions

Study procedures have been described previously [16]. Participants received oral metformin 850 mg (Galenicum Vitae, Barcelona, Spain) or matched placebo for 96 weeks. To improve gastrointestinal tolerability, treatment was initiated at 850 mg once daily, and increased to 850 mg twice daily after 4 weeks. For participants receiving dolutegravir, the dose remained at 850 mg once daily because dolutegravir increases metformin exposure and reduces clearance through inhibition of organic cation transporter 2 [17].

### Study Visits and Assessments

Study visits occurred at baseline, week 4, week 8 (if dose escalation was implemented), and weeks 24, 48, 72, and 96, with an additional post-treatment follow-up visit at weeks 120 and 144. Body weight was measured at each visit using calibrated scales, height was measured at baseline, and BMI was calculated at each visit as weight (kg) divided by height squared (m^2^). Waist and hip circumferences were measured using a flexible, nonstretchable tape at baseline, week 96, and week 144. Fasting blood samples, obtained after an overnight fast (≥8 h), were collected at baseline, week 96, and week 144 to assess metabolic parameters, including fasting plasma glucose, fasting insulin, HbA1c, HOMA-IR, and lipid profile. Serum growth differentiation factor 15 (GDF-15) levels were measured at baseline and at weeks 48, 96, and 144 using an ELISA (Bio-Techne; Minneapolis, MN, USA) according to the manufacturer's protocol.

Adverse events were assessed at each visit by clinical evaluation and laboratory testing and graded according to the Division of AIDS Table for Grading the Severity of Adult and Paediatric Adverse Events (version 2.1). Adverse reactions were defined as adverse events which were possibly, probably, or definitely causally related to metformin.

### Outcomes

Metabolic outcomes included between-group differences in mean change from baseline in body weight and BMI at week 96, corresponding to the end of the treatment period, with additional assessment at week 144 after treatment discontinuation. We also assessed the longitudinal trajectory of body weight and BMI from baseline to week 144 and the proportion of participants experiencing clinically relevant weight gain, defined categorically as an increase of 3% or more and 5% or more from baseline at week 96 and after treatment discontinuation.

Additional metabolic outcomes included changes in waist and hip circumference and changes from baseline to weeks 96 and 144 in fasting plasma glucose, HbA1c, fasting insulin, HOMA-IR, and lipid parameters. Serum GDF-15 was measured as an exploratory biomarker associated with metformin exposure and weight regulation [18]. Adverse events were classified by severity and causality as previously described.

### Statistical Analysis

As this was an exploratory pilot study, no formal sample size calculation was performed; the protocol specified a pragmatic target of 60 participants to be randomized. Accordingly, analyses of metabolic outcomes were considered hypothesis-generating.

Participant characteristics in the intention-to-treat population were summarized as absolute and relative frequencies for categorical variables and as medians with interquartile ranges for continuous variables. Analyses of metabolic efficacy outcomes, including body weight, and body mass index trajectories, were conducted in the per-protocol population (defined as all randomized participants who remained on assigned treatment through week 96); this population was selected for the primary efficacy analysis given the exploratory nature of the study and the high retention rate (87.5%). Safety analyses were performed in the safety population (all participants who received at least 1 dose of study medication).

Between-group differences in change from baseline to week 96 in body weight and BMI were estimated using linear regression models. To assess the evolution of weight and BMI across all study visits, we also used linear mixed-effects models with random intercepts to account for within-participant correlation over time. Fixed effects included treatment group, time (modeled as a continuous variable corresponding to study visit), and the treatment-by-time interaction to assess differences in trajectories between groups. Between-group differences after treatment discontinuation were assessed by comparing changes from week 96 to week 144 between groups using both linear regression models and linear mixed-effects models, as described above. Between-group differences in secondary metabolic parameters (glycemic indices, lipid parameters, and waist and hip circumferences) reported in the Supplementary material were estimated using analysis of covariance (ANCOVA), with the corresponding baseline value as covariate and treatment group as factor; results are reported as adjusted differences with 95% CIs and 2-sided *P* values. Prespecified exploratory correlation between changes in GDF-15 and changes in body weight was assessed using Pearson's correlation coefficient within each treatment group (n = 17 for metformin, n = 18 for placebo). All analyses were performed using R version 4.2.2. No imputation was performed for missing data; available-case analyses were used. All statistical tests were 2-sided, with a significance level of 0.05.

### Role of the Funding Source

The funder (Instituto de Salud Carlos III and European Union) and sponsor (La Paz University Hospital Research Foundation) had no role in the collection, data analysis, data interpretation, writing of the report, or the decision to submit the results for publication.

## RESULTS

**Figure 1.**
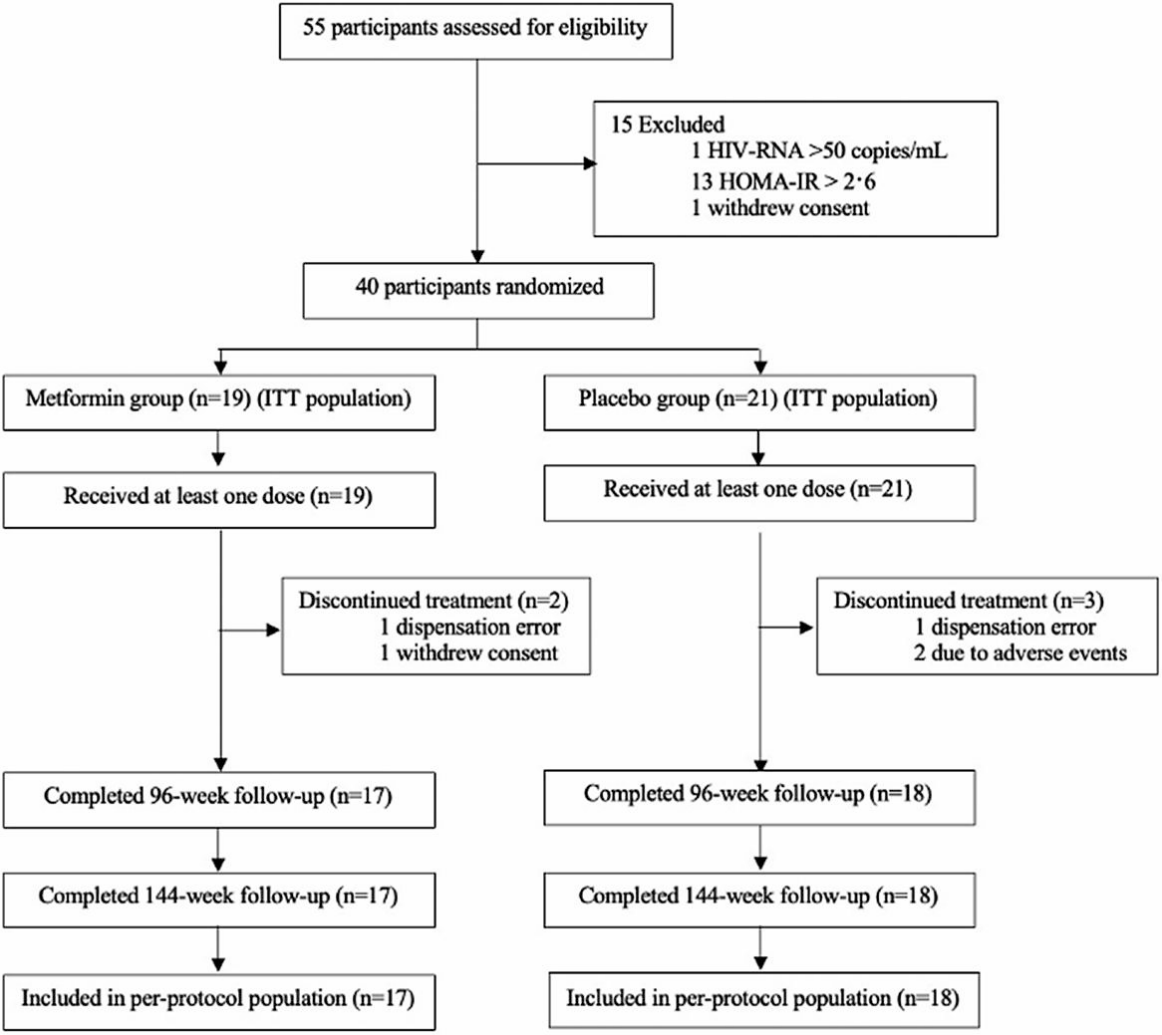
Trial profile.

Between 2 March and 2 October 2022, 55 individuals were screened, and 40 were randomly assigned to metformin (n = 19) or placebo (n = 21) (Figure 1). Enrolment was stopped at 40 participants because of slow recruitment in this single-center pilot study, below the protocol-specified pragmatic target of 60 participants. Five participants discontinued treatment before week 96 (metformin group: 1 dispensation error, 1 withdrew consent; placebo group: 1 dispensation error, 2 due to adverse events), and 35 participants (metformin n = 17; placebo n = 18) remained on allocated treatment through week 96 and comprised the per-protocol population. All of these participants completed the post-treatment follow-up until week 144.

Baseline characteristics were balanced between groups. Median age was 56.4 years (IQR 53.0–60.8), 12 (30%) participants were female, and 35 (87.5%) were White (Table 1). All participants had sustained virological suppression and preserved immune status at study entry. The majority of participants were receiving INSTI-based antiretroviral therapy, most commonly dolutegravir or bictegravir. Median baseline BMI was within the normal range in both groups, and no participants had obesity by study design. Most participants had either normal weight or were overweight, with similar BMI category distribution between treatment groups (Table 1).

**Table 1.** Baseline Characteristics of the Intention-to-Treat Population

	Metformin Group (n = 19)	Placebo Group (n = 21)
Chronological age (y)	56.5 (53–62.4)	56.2 (53.7–59.7)
Sex at birth (%)		
Female	7 (36.8)	5 (23.8)
Male	12 (63.2)	16 (76.2)
Ethnicity (%)		
White	17 (89.5)	18 (85.7)
Latin	2 (10.5)	3 (14.3)
Current smokers (%)	7 (36.8)	4 (19.0)
Mode of HIV transmission (%)		
Men who have sex with men	10 (52.6)	10 (47.6)
Heterosexual	6 (31.6)	2 (9.5)
Injection drug use	3 (15.8)	9 (42.9)
Nadir CD4 (cells/mm^3^)	311 (91–350)	304 (200–428)
CD4 count (cells/mm^3^)	777 (630.5–971)	729 (644–887)
Ratio CD4/CD8	1.1 (0.9–2.1)	1.2 (1.0–1.4)
Years since HIV diagnosis	22.5 (18.2–25.0)	26.6 (20.0–31.8)
INSTI regimen with DTG (%)	11 (57.9)	16 (76.2)
INSTI regimen with BIC (%)	6 (31.6)	2 (9.5)
Weight (kg)	71 (64–78.8)	74.9 (66.7–79.5)
BMI (kg/m^2^)	24.3 (22.9–25.8)	24.5 (22.0–26.0)
Underweight (<18.5)	0	1 (4.8)
Normal weight (18.5–<25)	12 (63.2)	10 (47.6)
Overweight (25–<30)	7 (36.8)	10 (47.6)
Waist circumference (cm)	92 (86–96)	91 (86–94)
Hip circumference (cm)	96.5 (93.2–102.8)	96 (94–100)
HOMA-IR	1.6 (1.3–2.3)	1.4 (1.1–2.5)
Fasting glucose (mg/dL)	88 (83.5–94.5)	83 (79–90)
HbA1c (%)	5.4 (5.3–5.6)	5.5 (5.3–5.7)
Total cholesterol (mg/dL)	179 (148.5–191)	177 (171–202)
LDL cholesterol (mg/dL)	91 (85–127.5)	109 (89–124)
HDL cholesterol (mg/dL)	45 (38–49.5)	51 (40–62)
ASCVD risk (%)	5.5 (3.2–11.4)	5.5 (4.6–11.8)

Data are presented as n (%) or median (IQR). Abbreviations: ASCVD, atherosclerotic cardiovascular disease; BIC, bictegravir; BMI, body mass index; DTG, dolutegravir; HbA1c, glycated hemoglobin; HOMA-IR, Homeostatic Model Assessment for Insulin Resistance; INSTI, integrase strand transfer inhibitor.

**Figure 2.**
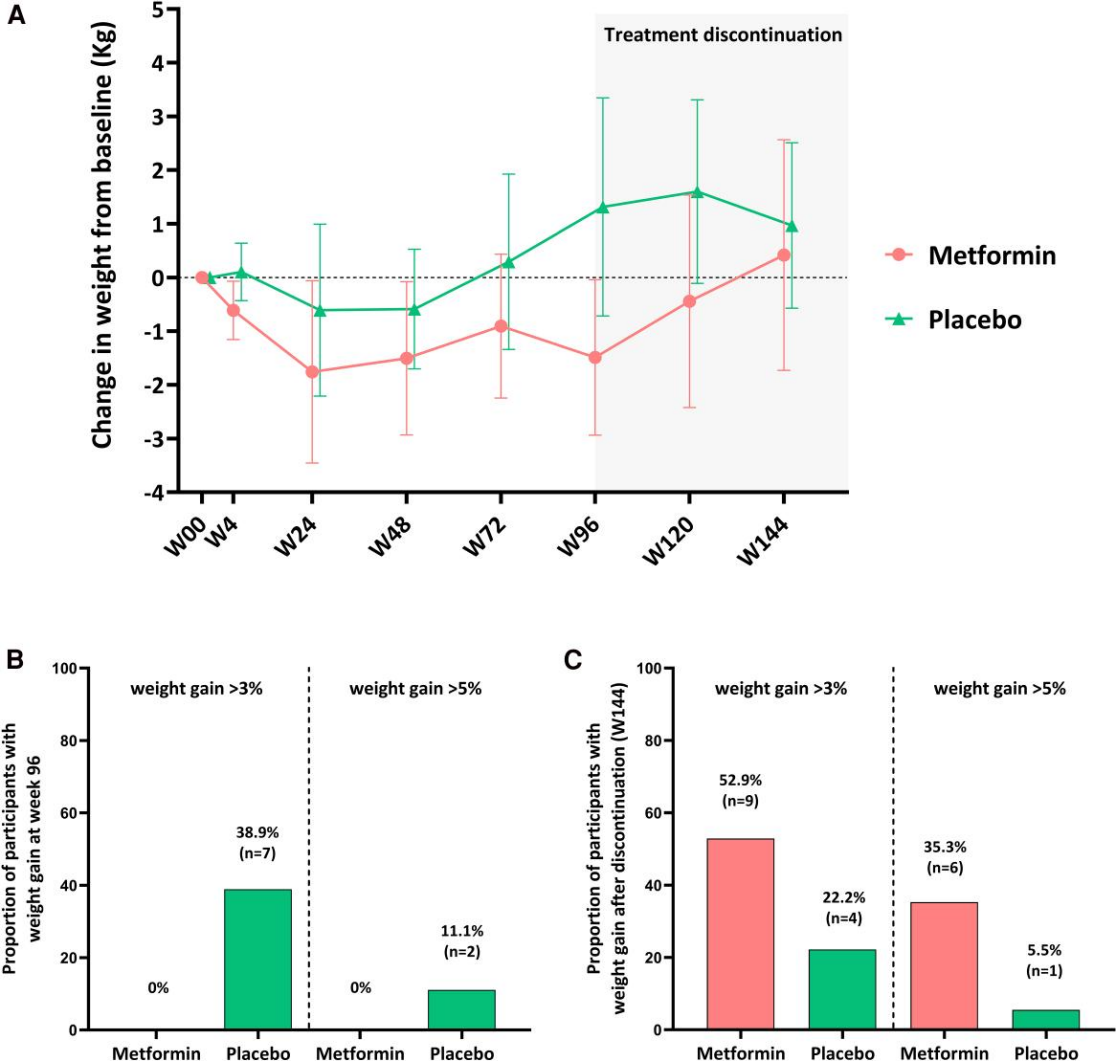
Longitudinal changes in body weight and categorical weight gain thresholds. *A*, Mean change in body weight from baseline to week 144 in the metformin and placebo groups; Error bars represent 95% CIs. *B*, Proportion of participants with ≥3% and ≥5% weight gain from baseline to week 96. *C*, Proportion of participants with ≥3% and ≥5% weight gain from week 96 to week 144.

During the treatment period, body weight trajectories diverged between groups (Figure 2*A*). At week 96, mean weight change from baseline was −1.5 kg (95% CI −2.9 to −0.03) in the metformin group and +1.3 kg (95% CI −0.7 to 3.3) in the placebo group, with a between-group difference of −2.8 kg (95% CI −5.2 to −0.4; *P* = .025). BMI followed a similar pattern, with a mean change of −0.5 kg/m^2^ (95% CI −0.9 to −0.02) in the metformin group and +0.4 kg/m^2^ (−0.2 to 1.1) in the placebo group; between-group difference of –0.9 kg/m^2^ (95% CI –1.7 to –0.1; *P* = .022) (Supplementary Figure 1).

Linear mixed-effects models with random intercepts confirmed these trends over the entire treatment follow-up period. In the reference group (placebo), body weight increased significantly over time (*β* = .011 kg/week; *P* = .034), whereas the metformin group showed attenuation of this increase. A significant treatment-by-time interaction was observed, with weight decreasing by 0.021 kg/week in the metformin group compared with placebo (*β* = −.021 kg/week; 95% CI −0.036 to −0.006; *P* = .007). Similar findings were observed for BMI (*β* = −.007 kg/m^2^ per week; 95% CI −0.012 to −0.002; *P* = .006).

These findings were supported by categorical analyses (Figure 2*B* and *2C*). During treatment period (baseline to week 96), no participants in the metformin group experienced clinically relevant weight gain (≥3% or ≥5%), whereas 38.9% (n = 7) and 11.1% (n = 2) of placebo-treated participants experienced ≥3% and ≥5% weight gain, respectively.

After treatment discontinuation, weight values in the metformin group approached baseline levels (Figure 2*A*). Between weeks 96 and 144, participants previously assigned to metformin experienced greater weight gain than those receiving placebo (+1.9 kg [95% CI 0.2 to 3.5] vs −0.3 kg [95% CI −1.9 to 0.3]; mean difference of 2.2 kg [95% CI 0.05 to 4.5]; *P* = .045). BMI showed a similar pattern, with an increase of approximately 0.7 kg/m^2^ in the metformin group and −0.1 kg/m^2^ in the placebo group; mean difference of 0.8 kg/m^2^ (95% CI 0.03 to 1.5; *P* = .041) (Supplementary Figure 1). In longitudinal analyses using linear mixed-effects models, we also observed a significant treatment-by-time interaction, indicating that participants allocated to metformin experienced greater weight gain (*β* = .047 kg/week; 95% CI 0.009 to 0.084; *P* = .013) and an increase in BMI (*β* = .016 kg/m^2^ per week; 95% CI 0.004 to 0.028; *P* = .010) over the entire post-treatment period. In the categorical analysis the proportion of participants with clinically relevant weight gain after treatment discontinuation in the metformin group increased to 52.9% (n = 9) for the ≥3% threshold and 35.3% (n = 6) for the ≥5% threshold (Figure 2*C*).

**Figure 3.**
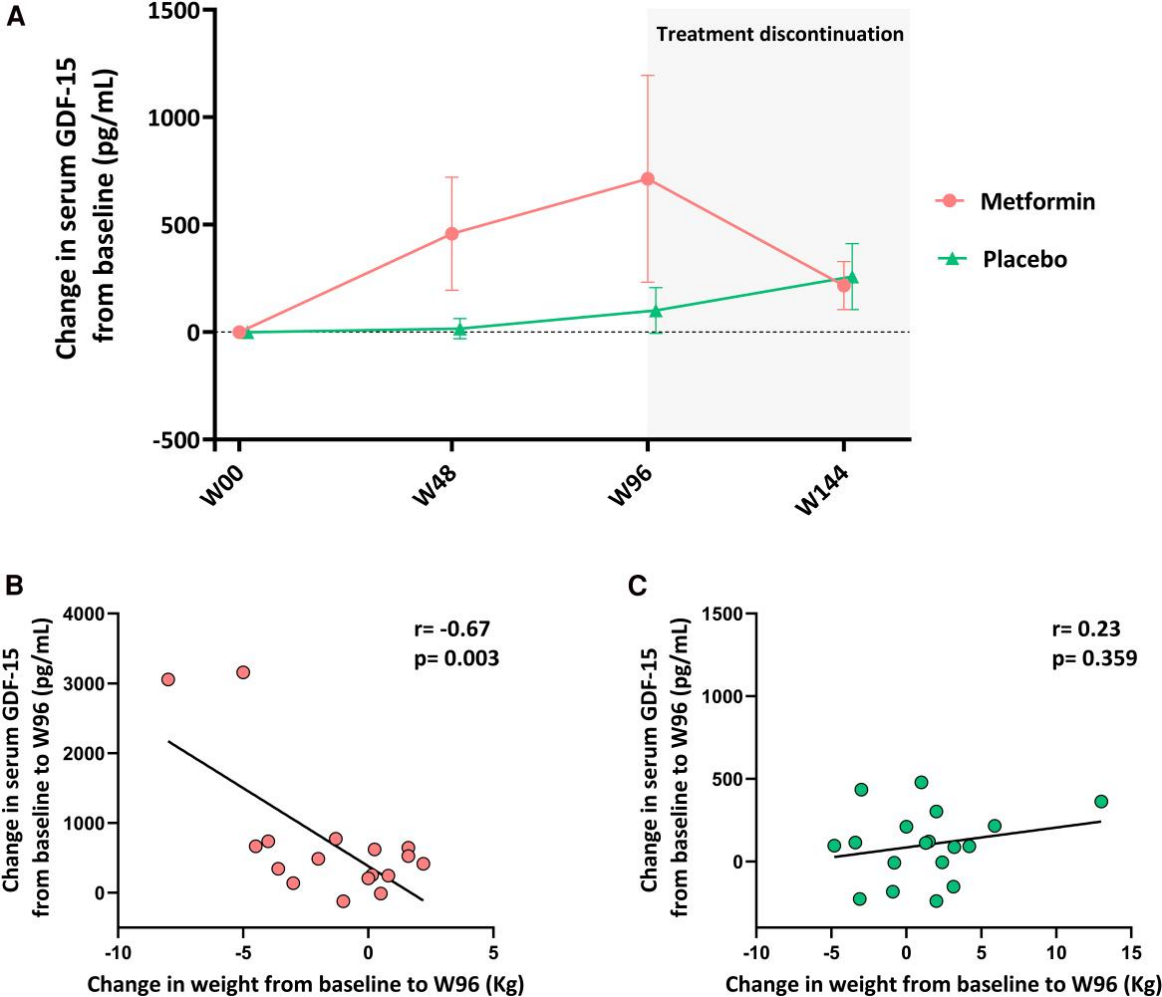
Serum GDF-15 trajectories and correlation with body weight change. *A*, Mean change in serum GDF-15 concentrations from baseline to week 144 in the metformin and placebo groups; Error bars represent 95% CIs. *B, C*, Correlations between change in serum GDF-15 and change in body weight from baseline to week 96 in the metformin (*B*) and placebo groups (*C*). Pearson correlation coefficients and *P* values are shown.

Serum GDF-15 concentrations increased during the treatment period in participants receiving metformin, with a progressive rise from baseline to week 96 (*P* < .001), followed by a significant decline after treatment discontinuation toward week 144 (*P* < .001; Figure 3*A*). At week 96, the mean change from baseline in GDF-15 was significantly greater with metformin than with placebo (489.9 pg/mL vs 104.4 pg/mL; *P* < .001). By contrast, GDF-15 concentrations remained relatively stable over time in the placebo group. Changes in GDF-15 correlated inversely with changes in body weight in the metformin group (*r* = −0.67; *P* = .003), whereas no association was observed in the placebo group (*r* = 0.23; *P* = .359; Figure 3*B* and *3C*).

No significant between-group differences were observed in other metabolic parameters (fasting glucose, fasting insulin, glycated hemoglobin, and HOMA-IR) either during the intervention period or after treatment discontinuation. Likewise, no meaningful differences were observed in waist or hip circumference or lipid parameters (Supplementary Tables 1 and 2).

Safety outcomes were as previously reported in the primary trial analysis [16]. Overall, adverse event frequency was similar between groups, and no serious adverse events were attributed to metformin. Gastrointestinal adverse events were generally mild and self-limited; diarrhea occurred in 4 participants (21%) in the metformin group, and 1 participant required dose adjustment because of persistent symptoms. No participants discontinued treatment because of metformin-related adverse events.

No significant between-group differences were observed in laboratory safety parameters, including CD4+ T-cell count, serum creatinine, and liver enzymes. All participants maintained virological suppression throughout follow-up. Detailed laboratory safety data are reported in the primary trial analysis [16].

## DISCUSSION

In this randomized, placebo-controlled pilot trial of nondiabetic, nonobese, older PWH receiving predominantly INSTI-based ART, metformin attenuated progressive weight gain over 96 weeks compared with placebo. Participants assigned to metformin experienced modest weight loss, whereas those receiving placebo gained weight. After treatment discontinuation at week 96, body weight in the metformin group returned toward baseline by week 144, indicating that the observed effect was exposure-dependent and may require ongoing treatment to be maintained.

Notably, this was, to our knowledge, one of the first randomized trials to administer metformin for 96 weeks in participants selected to exclude obesity, prediabetes, diabetes, and insulin resistance. Whereas long-term metformin studies in HIV-negative populations have largely focused on individuals with metabolic risk or established cardiometabolic disease, METFORAGING evaluated metformin in a metabolically normal population, enabled by its primary design as a biological aging intervention.

Previous studies evaluating metformin in PWH have been limited by short duration and nonrandomized designs. In the LILAC study [19], a single-arm, nonrandomized pilot trial, short-term metformin (12 weeks) was associated with modest weight loss that reversed after discontinuation. Our study demonstrates suppression of weight gain over a substantially longer follow-up within a randomized, placebo-controlled design. The consistent observation across both studies that weight returned toward baseline after stopping metformin suggests that the effect on body weight is reversible and dependent on continued exposure.

Metformin also increased serum GDF-15 levels, and changes in body weight correlated inversely with GDF-15 dynamics only in the metformin group. These associations were observed in a population without insulin resistance at baseline and in the absence of changes in glycemic parameters during follow-up, supporting a weight-regulating effect independent of glycemic control. Similar findings were reported in the LILAC study [19], in which short-term metformin administration in nondiabetic PWH was associated with modest weight loss and inverse correlations between weight and GDF-15 levels, with both parameters returning toward baseline after treatment discontinuation.

Data from the CAMERA trial in nondiabetic HIV-negative individuals with cardiovascular disease also showed inverse correlations between weight change and GDF-15 among metformin recipients, with no similar pattern in the placebo group [20]. GDF-15 acts via the hindbrain-restricted GFRAL receptor involved in feeding regulation, and elevations in circulating GDF-15 have been associated with reduced appetite and lower energy intake [9]. The directionality and reversibility observed across the CAMERA, LILAC, and our study support a potential link between metformin-induced changes in GDF-15 and weight regulation.

Metformin was well tolerated over 96 weeks, with a safety profile comparable to placebo, despite the absence of a metabolic indication for treatment. No treatment discontinuations or serious adverse events were attributed to metformin, consistent with earlier studies in PWH, including the LILAC trial [21], and the MAVMET trial [22].

The study has limitations typical of a pilot trial. The small sample size reduces precision and limited our ability to explore heterogeneity of treatment effect or to undertake extensive multivariable adjustment. Participants were predominantly White and nonobese, so generalizability to PWH with obesity, and to more diverse ethnic backgrounds is uncertain. An imbalance in INSTI regimen distribution between groups (bictegravir 31.6% in metformin vs 9.5% in placebo) was observed; since participants receiving dolutegravir received a reduced metformin dose because of pharmacokinetic interactions, this differential dosing may have influenced the magnitude of effect. The single-center design may also limit external validity. Weight was a secondary outcome and analyses were exploratory. Finally, body-composition imaging was not performed, precluding assessment of changes in fat mass, and distribution.

In summary, metformin attenuated progressive weight gain in nondiabetic, nonobese older PWH receiving contemporary ART, and the effect appeared reversible after treatment discontinuation. The favorable safety profile over nearly 2 years supports further evaluation of metformin as a pragmatic, scalable approach to weight management in HIV care. Future studies should include PWH with overweight or obesity and broader demographic representation and be adequately powered to assess efficacy and durability.

## Supplementary Material

ofag489_Supplementary_Data

## Supplementary Data

Supplementary materials are available at *Open Forum Infectious Diseases* online. Consisting of data provided by the authors to benefit the reader, the posted materials are not copyedited and are the sole responsibility of the authors, so questions or comments should be addressed to the corresponding author.
