# Supplementary material for: Metformin and Weight Changes in Non-diabetic Older People With HIV (METFORAGING): A Double-Blind, Randomized, Placebo-Controlled, Pilot Trial
Source: Open Forum Infect Dis. 2026 Aug 6;13(8):ofag489. doi: 10.1093/ofid/ofag489 (PMC13479022; doi:10.1093/ofid/ofag489)
Supplement: Supplementary file 1 [file ofag489_supplementary_data.docx]

**Supplementary material**

### **Supplementary Table S1. Changes in metabolic parameters from baseline to week 96 by** **treatment group**

| **Parameter** | **Adj. diff (M-P)** | **95%CI low** | **95%CI high** | ***p-*value*** |
| --- | --- | --- | --- | --- |
| Fasting glucose, mg/dL | 3.80 | -1.90 | 9.50 | .184 |
| Fasting insulin, μU/mL | -1.57 | -6.41 | 3.26 | .512 |
| HbA1c, % | -0.01 | -0.12 | 0.10 | .854 |
| HOMA-IR | -0.27 | -1.33 | 0.79 | .606 |
| Total cholesterol, mg/dL | -12.76 | -26.20 | 0.69 | .062 |
| HDL cholesterol, mg/dL | 0.18 | -3.99 | 4.35 | .931 |
| LDL cholesterol, mg/dL | -13.42 | -28.18 | 1.34 | .073 |
| Triglycerides, mg/dL | 0.28 | -22.24 | 22.80 | .980 |
| Waist circumference, cm | -1.65 | -5.34 | 2.04 | .368 |
| Hip circumference, cm | -1.69 | -5.34 | 1.96 | .352 |

### *ANCOVA model adjusted difference reported as Metformin minus Placebo with 95% CI and two-sided *p*-value.

Abbreviations: CI, confidence interval; HbA1c, glycated hemoglobin; HDL, high-density lipoprotein; HOMA-IR, Homeostatic Model Assessment for Insulin Resistance; LDL, low-density lipoprotein; M, metformin; P, placebo.

### **Supplementary Table S2. Changes in metabolic parameters from week 96 to week 144 by treatment group**

| **Parameter** | **Adj. diff (M-P)** | **95%CI low** | **95%CI high** | ***p*-value*** |
| --- | --- | --- | --- | --- |
| Fasting glucose, mg/dL | 0.57 | -5.55 | 6.69 | .851 |
| Fasting insulin, μU/mL | 1.43 | -0.95 | 3.80 | .230 |
| HbA1c, % | 0.08 | -0.05 | 0.20 | .218 |
| HOMA-IR | 0.40 | -0.09 | 0.90 | .109 |
| Total cholesterol, mg/dL | 8.15 | -7.13 | 23.43 | .286 |
| HDL cholesterol, mg/dL | -1.52 | -6.81 | 3.77 | .563 |
| LDL cholesterol, mg/dL | 6.36 | -6.26 | 18.99 | .312 |
| Triglycerides, mg/dL | 12.28 | -4.81 | 29.38 | .153 |
| Waist circumference, cm | 1.13 | -1.67 | 3.94 | .417 |
| Hip circumference, cm | -0.94 | -3.88 | 1.99 | .518 |

### *ANCOVA model adjusted difference reported as Metformin minus Placebo with 95% CI and two-sided *p*-value.

Abbreviations: CI, confidence interval; HbA1c, glycated hemoglobin; HDL, high-density lipoprotein; HOMA-IR, Homeostatic Model Assessment for Insulin Resistance; LDL, low-density lipoprotein; M, metformin; P, placebo.

**Supplementary Figure 1. Longitudinal changes in body mass index (BMI).**

### Mean change in BMI from baseline to week 144 in the metformin and placebo groups. The shaded area indicates the post-treatment period after discontinuation at week 96. Error bars represent 95% CIs
